# Growing-Related Changes in Arterial Properties of Healthy Children, Adolescents, and Young Adults Nonexposed to Cardiovascular Risk Factors: Analysis of Gender-Related Differences

**DOI:** 10.1155/2016/4982676

**Published:** 2016-02-18

**Authors:** S. Curcio, V. García-Espinosa, M. Arana, I. Farro, P. Chiesa, G. Giachetto, Y. Zócalo, D. Bia

**Affiliations:** ^1^Physiology Department, Faculty of Medicine, CUiiDARTE, University of the Republic, General Flores 2125, 11800 Montevideo, Uruguay; ^2^Clínica Pediátrica “C”, Pereira-Rossel Hospital Centre, Faculty of Medicine, University of the Republic, Bulevar Artigas 1550, 11600 Montevideo, Uruguay; ^3^Pediatric Cardiology Service, Pereira-Rossell Hospital Centre, ASSE, Bulevar Artigas 1550, 11600 Montevideo, Uruguay

## Abstract

The aims of our work were to determine normal aging rates for structural and functional arterial parameters in healthy children, adolescents, and young adults and to identify gender-related differences in these aging rates.* Methods*. 161 subjects (mean: 15 years (range: 4–28 years), 69 females) were studied. Subjects included had no congenital or chronic diseases, nor had they been previously exposed to traditional cardiovascular risk factors. Arterial parameters assessed were (1) central blood pressure (BP) and aortic pulse wave analysis, (2) arterial local (pressure-strain elastic modulus) and regional (pulse wave velocity, PWV) stiffness, and (3) arterial diameters and carotid intima-media thickness (CIMT). Simple linear regression models (age as the independent variable) were obtained for all the parameters and the resulting rates of change were compared between genders.* Results*. No gender-related differences were found in mean values of arterial structural and functional parameters in prepubertal ages (4–8 years), but they started to appear at ~15 years. Boys showed a greater rate of change for central systolic BP, central pulse pressure, CIMT, and carotid-femoral PWV.* Conclusion.* Gender-related differences in arterial characteristics of adults can be explained on the basis of different growing-related patterns between boys and girls, with no existing differences in prepubertal ages.

## 1. Introduction

In adults, age-related changes in structural and functional arterial properties have been well described and are jointly known as* vascular aging* (VA) [1]. These changes include arterial stiffening, luminal dilatation, and wall thickening [2] and are known to have a heterogeneous distribution between central and peripheral arteries [3]. As VA has been shown to accelerate in association with several pathological conditions and with cardiovascular risk factors (CRF) exposure (leading to target organ damage (TOD) and increased cardiovascular risk) [2, 4], the concept of* early vascular aging* (EVA) has been recently proposed and used in order to discriminate between normal and pathological VA [5]. The importance of EVA lies in its usefulness as an indicator of cumulative arterial damage and increased risk of cardiovascular events. Therefore, identifying subjects with a burden of components of the so-called EVA syndrome [5] in early stages could allow designing proper interventions to prevent and reduce vascular alterations, thus minimizing cardiovascular risk. Supported by the fact that the process of arterial impairment begins during childhood and by existing evidence of positive outcomes of intervention studies in pediatric populations [6–8], it has been suggested that preventing and controlling EVA should start in early life [9].

On the other hand, the existence of gender-related differences in arterial properties and cardiovascular risk is a well-known and described fact for adults [10–13]. At least in theory, sex-related differences in arterial behavior in adults could be explained on the basis of differences in growth-related vascular changes between boys and girls. However, whether these differences exist since birth or if they appear with growth in children and adolescents is still a controversial issue. In the latter case, information would also be lacking about the ages in which those differences can start being detected, for which parameters and in what magnitude. Moreover, information is lacking about normal VA in early ages. Describing normal VA patterns for these populations would allow distinguishing between normal VA and EVA and also determining those ages in which sex-distinction is needed for reference values utilization.

In this context, the aims of this work were (1) to determine normal growth-related changes for several vascular structural and functional parameters in a healthy and nonexposed to CRF population of children, adolescents, and young adults and (2) to identify the presence of gender-related differences in growth rates for different arterial properties.

## 2. Methods

### 2.1. Study Population

CUiiDARTE Center and Project is a Uruguayan Interdisciplinary University Program for Arterial Disease Early Diagnosis advocated in children and adults, supported by the Public Health Minister [14, 15]. Studied individuals were derived for arterial evaluation by CUiiDARTE Project medical staff. Subjects included had no congenital, chronic, or infectious diseases at the time of the study, nor had they been previously exposed to traditional CRF as hypertension, smoking, obesity, diabetes, dyslipidemia, physical inactivity, and premature family history of cardiovascular disease.

All studies were approved by the Institutional Ethics Committee, and written informed consent was obtained prior to the examination.

### 2.2. Medical Interview and Anthropometrics

Before vascular evaluation a clinical interview was conducted in order to assess CRF exposure. Subjects' weight and height were measured. Body mass index (BMI) was calculated as the weight-to-squared height ratio and, for underaged subjects, converted into percentiles/*z*-scores using the World Health Organization reference values. Children and adolescents with a BMI >97th percentile and/or a BMI *z*-score >2, for age and sex, and adults with a BMI >30 Kg/m^2^ were excluded from the study. Children and adolescents with known arterial hypertension and/or with blood pressure (BP) levels >95th percentile for age, sex, and height were excluded. Adults with BP ≥ 140/90 mmHg were also excluded.

Finally, 161 healthy subjects nonexposed to cardiovascular risk factors (mean age: 15 years (range: 4–28 years), 69 females) were included in the present work.

### 2.3. Study Protocol

Prior to the study, all individuals had been for at least 4 hours fasting and took a 15-minute rest in supine position, in a calm environment with a controlled room temperature (21–23°C). During the examination, brachial BP and heart rate (HR) measurements were performed at fixed intervals of 8–10 minutes (HEM-433INT; Omron Healthcare Inc., Illinois, USA).

#### 2.3.1. Arterial Stiffness

Pulse wave velocity (PWV) was measured to assess aortic (carotid-femoral PWV, cfPWV) and upper limb (carotid-radial PWV, crPWV) arterial stiffness, by applanation tonometry (SphygmoCor, AtCor Medical, Australia) [21]. Pulse wave travel distance was assessed using surface tape measurements. For cfPWV, the pulse wave path length was measured as the distance between carotid and femoral sampling sites, and for crPWV it was measured as the distance between radial sampling site and the sternal notch. In order to obtain “real” cfPWV [22], calculated cfPWV was multiplied by a scaling factor of 0.8. The resulting PWV values consisted of the mean of three different measurements and were only considered valid if standard deviation (SD) between measurements was <10%. Additionally, seeking to normalize cfPWV values by BP levels, *β*PWV Index was calculated as follows: Ln(SBP/DBP) × 2.11 × cfPWV2/PP, Ln being the natural logarithm and SBP, DBP, and PP the systolic, diastolic, and pulse pressure level [23].

Pressure-strain elastic modulus (EM) was calculated as follows: EM = PP/((SD − DD)/DD), where SD and DD are systolic and diastolic arterial diameters. Central PP was used to calculate common carotid artery (CCA) EM and peripheral PP to calculate common femoral artery (CFA) EM. Vascular ultrasound (6–13 MHz linear array probe, M-Turbo, SonoSite Inc., WA, USA) was used to obtain 30-second longitudinal image sequences of left and right CCA and CFA, which were stored for off-line analysis. Beat-to-beat diameter waveforms were obtained using automatic border detection software. SD and DD were quantified as the average of at least twenty beats [24]. Local stiffness was normalized by BP calculating *β* Index as follows: Ln(SBP/DBP)/((SD − DD)/DD).

#### 2.3.2. Intima-Media Thickness

CCA intima-media thickness (CIMT) was assessed in the proximal centimeter from the carotid bifurcation, using ultrasound system and specific software [25, 26]. CIMT was measured in the “posterior” wall at the moment corresponding to the end DD, and its value was the mean resulting from at least 10 measurements.

#### 2.3.3. Central Aortic Pressure and Wave Reflection Parameters

In order to assess central aortic pressure and reflection parameters, radial artery pressure waveforms were recorded using applanation tonometry (SphygmoCor, AtCor Medical, Australia). After 20 seconds of recording, waveforms acquired were calibrated and averaged, and a generalized transfer function was used to synthesize the corresponding central aortic pressure waveform [27].

Augmentation index (AIx), augmented pressure (AP), and subendocardial viability ratio (SEVR) were derived from the aortic pressure waveform using pulse waveform analysis (PWA). Considering that AIx is influenced by HR and height, an index normalized for a HR of 75 bpm was used (AIx@75) [28], and it was afterwards normalized by height calculating an AIx@75-to-height ratio.

### 2.4. Data Analysis and Statistics

Data are expressed as the mean ± standard deviation. Data analysis was performed using SPSS software (SPSS Inc., Illinois, USA). To study the relationship between age and arterial parameters, subjects were grouped by gender and classified in three age categories: <8, 8–14, and ≥15 years. Continuous variables were compared using tests according to their statistical distribution.

Reproducibility of the ultrasound technique and its derived variables was tested by analyzing intra- and interobserver variability (assessed as variation coefficient (VC) and intraclass correlation coefficient (ICC)). In this case, two trained observers (SC and VGE) were compared. Parameters analyzed were SD, DD, and CIMT. Overall intraobserver VC were as follows: SC (DD = 0.66%, SD = 0.55%, and IMT = 2.05%) and VGE (DD = 1.2%, SD = 0.56%, and IMT = 2.69%). Intraobserver ICC (95% confidence intervals expressed) between measurement times (*T*1 and *T*2) was 0.978 (0.910–0.994). Interobserver ICC was as follows: SD = 0.919 (0.811–0.965), DD = 0.917 (0.808–0.964), and IMT = 0.985 (0.944–0.996).

Age-related changes in arterial parameters were also explored by obtaining simple linear regression models for each gender. Gender-related differences in aging patterns were then investigated by comparison of the slopes of the corresponding models obtained, analyzing the interaction between age and sex as covariables (Table 2). An exception was made with the model obtained for AIx@75. As it was previously described that values for this parameter start to increase at ~20 years of age [29], to accomplish a good analysis in this case we limited the studied population to those ages.

The models for the parameters showing a significant correlation with age were used to calculate the predicted mean values at 4 and 18 years of age, and afterwards the average relative change expected with growing from 4 to 18 years was calculated (Table 3). The 95% confidence intervals (CI) expressed in Table 3 were calculated from the 95% CI obtained for the slope of each model. For every analysis performed, a *p* value < 0.05 was considered significant.

## 3. Results

No gender differences existed in age, height, and weight, except for the elder group (≥15 years), where the expected physiological differences determined that males were significantly heavier and taller than females (Table 1).

### 3.1. Peripheral and Central Arterial Pressure

No gender-related differences were found in peripheral diastolic BP (pDBP), with independence of the age group. The lower and middle age groups showed no difference in peripheral systolic BP (pSBP). As was expected, in the elder group pSBP was significantly higher in males than in females. Peripheral PP (pPP) did not show difference between genders in the lower age group but was statistically different for the elder group. For the middle age group it was higher in boys, but with no statistic significance.

Similar results were found for aortic pressure levels. Central systolic BP (cSBP) was significantly higher for boys in the elder age group (107 mmHg versus 99 mmHg, *p* < 0.001). Central PP (cPP) reached the significance in the middle age group, maintaining this difference for the elder group, being also higher in boys (42 mmHg versus 31 mmHg, *p* < 0.001; Table 1).

### 3.2. Wave Reflection Parameters

AIx@75 was significantly higher in females starting in the middle age group and maintained this difference for the elder group (2.6% versus −3.9%, *p* < 0.001), showing a greater influence of wave reflections in central BP in females since early ages (Table 1). This difference remained significant after adjusting AIx@75 by height. SEVR was found to be lower in girls for all the age groups, but this difference did not reach the statistic significance.

### 3.3. Arterial Diameters and CIMT

Arterial diameters were greater for males than for females. In the CCA, the difference was significant for the elder group, whereas in the CFA it was significant in the middle and elder age groups (Table 1). Mean values of CIMT were found to be higher in males, only reaching the statistic significance for the elder age group (0.497 mm versus 0.459 mm, *p* = 0.04; Table 1).

### 3.4. Local and Regional Arterial Stiffness

In the middle and elder age groups, males showed higher local arterial stiffness (EM) for CCA, whereas no gender-related differences were found for CFA. The differences in CCA EM were however found to be dependent on BP levels for the lower and middle age groups, since when *β* Index was calculated no gender-related differences in CCA stiffness were found (Table 1). However, for the elder group the difference was found to be only partially pressure-dependent, suggesting that boys have higher intrinsic wall stiffness than girls since ~15 years.

Regional arterial stiffness in the upper limb (crPWV) showed no gender-related difference for the different age groups, whereas cfPWV was found to be higher in males, reaching the statistic significance for the elder group (6.6 m/s versus 5.6 m/s, *p* < 0.001). This difference was still found when cfPWV levels were normalized by BP (*β*PWV Index). These results show that no gender-related differences exist in regional arterial stiffness for muscular arteries, but they can be found for elastic arteries, such as the aorta, starting at ~15 years.

### 3.5. Gender Differences in Growing-Related Changes in Arterial Structural and Functional Parameters

crPWV did not show a significant correlation with age. The remaining parameters showed a significant linear and positive correlation with age, except for AIx@75, which had an inverse correlation with age (Table 2).

cSBP was found to have a steeper increase with age in males than in females (1.614 mmHg/year versus 0.902 mmHg/year, *p* < 0.001). A greater difference was found for cPP (1.135 mmHg/year in males versus 0.285 mmHg/year in females, *p* < 0.001), indicating that in males cPP increases more rapidly with age. These results translate into a ~28.9% increase in cSBP for boys versus ~15.6% for girls from 4 to 18 years (Table 3), whereas for cPP the gender difference in relative increase was even greater (male: ~72.6% versus female: ~16.4%).

**Figure 1:**
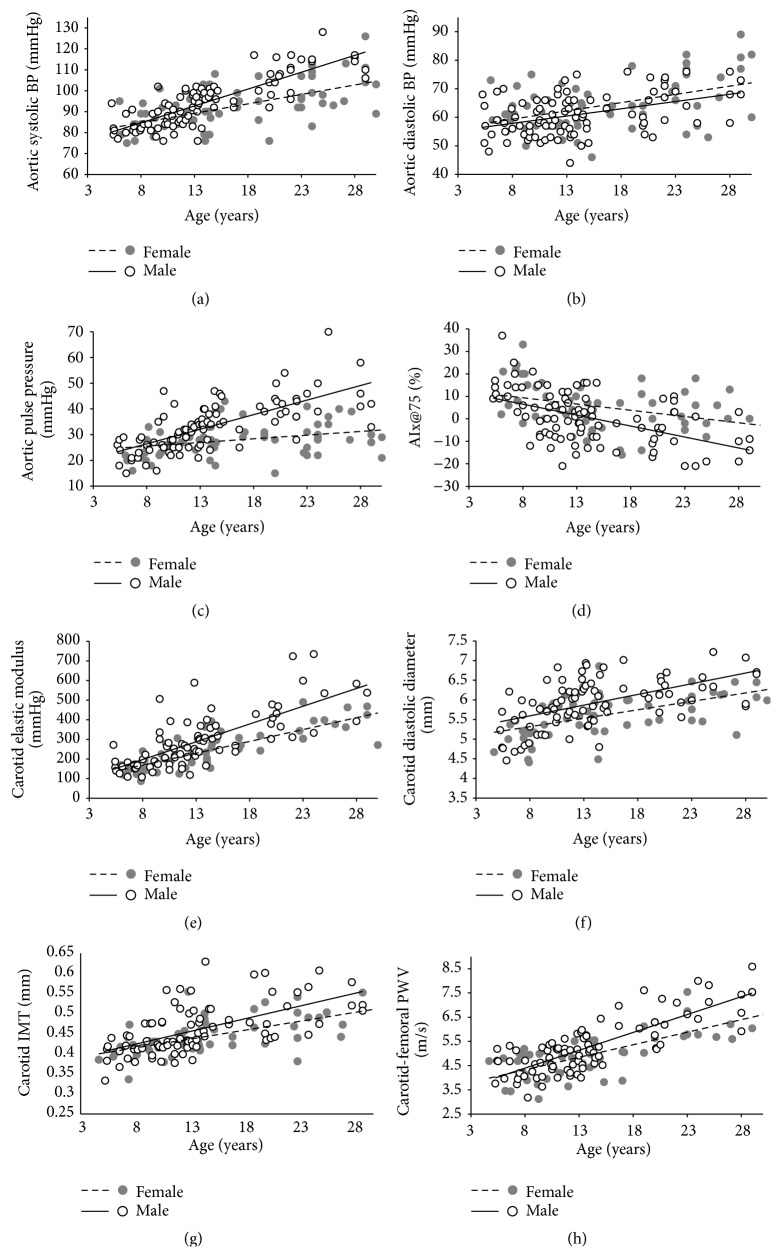
Regression plots for structural and functional arterial parameters. BP, blood pressure; AIx@75, augmentation index; IMT, intima-media thickness; PWV, pulse wave velocity.

AIx@75 also showed more pronounced age-related changes in males (Figure 1(d)), but in this case with no statistic significance. However, the existing difference determines that for boys the relative decrease in AIx@75 from 4 to 18 years is almost twice than for girls (~153% versus ~88%, Table 3).

No significant gender-related differences were found in the rate of change for CCA diameters, whereas for CIMT a significant and higher rate of change was found for males (0.006 mm/year versus 0.004 mm/year, *p* = 0.04; Table 2).

Local arterial stiffness, evaluated through EM, showed significant gender differences in age-related changes only for the CCA, indicating that males have a greater rate of increase in CCA stiffness with age (Table 2, Figure 1(e)). Moreover, when normalized by BP levels (CCA *β* Index), this gender-related difference could still be found, suggesting that the most marked increases in CCA stiffness are independent of the higher levels of BP in males (Table 2). This determines an important difference in the predicted relative increase for local stiffness from 4 to 18 years (~24.2% versus ~7.3%, Table 3).

Males showed a steeper increase in aortic stiffness (0.147 m/s/year versus 0.103 m/s/year, *p* = 0.018; Table 2, Figure 1(h)). The *β*PWV Index also showed a greater changing rate for males (Table 2). These results indicate that boys present more pronounced age-related changes in intrinsic stiffness (pressure-independent) of the aortic wall, resulting in an average increase of ~54% versus ~36.9% for girls from 4 to 18 years.

## 4. Discussion

To our knowledge, this is the first study characterizing gender-related differences in arterial properties aging rates as an integrated approach (including both structural and functional parameters) in healthy children, adolescents, and young adults free of traditional CRF exposure. From the analysis of our results, five findings are of major relevance:

1. During aging of healthy and nonexposed to CRF children, adolescents, and young adults, several changes can be found in both structural and functional arterial properties (Table 2). As the studied population was free of classic CRF exposure, our results suggest that these aging patterns could be explained on the basis of physiological changes due to development and growth in early ages.
2. In prepubertal ages (4–8 years), no gender-related differences can be found for arterial characteristics (Table 1).
3. The aging rates described determine that relative changes are lower for structural (arterial diameters and thickness) than functional and hemodynamic (aortic PP, arterial stiffness) parameters (Table 3).
4. These growing-related changes occur at a higher rate for boys than for girls (Table 2), suggesting the existence of an accelerated aging rate for males.
5. Jointly analyzed, the mentioned differences determine that (1) gender-related differences in pressure-dependent arterial stiffness can start being detected between 8 and 14 years and (2) since postpubertal ages gender-related differences in structural parameters can be evidenced, and arterial stiffness differences imply intrinsic wall changes, not only being determined by BP differences (Table 1).

### 4.1. Growing-Related Changes in Arterial Functional and Structural Parameters

Although several studies have characterized aging-related changes in vascular parameters for pediatric populations, little information is available about specific VA rates and the expected normal changes for these ages, even less utilizing an integrated approach as ours.

However, the tendencies shown by our results are similar to those found in other studies. For example, Hayward and Kelly [30] reported a marked age-related increase in central systolic and diastolic pressure and PP between the first two decades of life, as well as a decrease in AIx@75. This decrease in the influence of wave reflection on aortic augmentation is known to be highly dependent on the growth in height in early ages, as the wave reflection sites get progressively farther from the heart.

For structural parameters as luminal diameters and CIMT, several authors found significant increases with age in children and adolescents [20, 31–33]. Moreover, Engelen et al. [33] reported similar changing rates in CIMT for males (0.0052 mm/year) and females (0.0049 mm/year) to the ones we found in our population (0.006 mm/year and 0.004 mm/year, resp.). Whereas Ishizu et al. [32] reported a greater changing rate in CIMT (0.009 mm/year), their study included subjects with ages ranged between 5 and 14 years, and when we adjusted our regression models to a population limited to these ages a more similar rate of change could be found (0.008 mm/year, *r* = 0.463, *p* < 0.001). A possible explanation of these findings is that growing-related changes are not necessarily lineal but have differential patterns between smaller age ranges.

Age-related changes in local and regional stiffness parameters are also widely described for the first decades of life [16, 20, 34, 35]. Senzaki et al. [35] performed invasive measurements of aortic EM, finding a similar changing rate of ~10 mmHg/year in contrast to an average 14 mmHg/year found for carotid EM in our population. Reusz et al. [16] reported a strong correlation between age and cfPWV in a large multicentric study including more than 1000 children and adolescents.

Although most of the authors reporting reference values for arterial properties in children, adolescents, and young adults did not report the respective regression models, we estimated and compared them with ours by using the mean values provided for each age (Table 4).

### 4.2. Gender-Related Differences in Vascular Aging

In males there is an accelerated rate of change for most of the vascular parameters studied. Even though previous studies did not analyze the integral (structural and functional) evolution of arterial changes, consistently with our findings, most of the authors investigating gender-related differences in arterial structural and functional parameters could not find them in ages below ~10 years but reported important differences since pubertal ages, thus suggesting the existence of differential aging patterns related to sex.

#### 4.2.1. Differences in Hemodynamic Parameters

We found that boys present a greater rate of increase in cSBP and cPP. Since the presence of elevated central systolic pressure and PP determines an increase in cardiovascular risk, this phenomenon could be on the basis of the higher risk existing in males since early ages. In agreement with our findings, Hayward and Kelly [30] had similar results in their study, reporting a higher cPP in boys since the first decade of life. Recently, Elmenhorst et al. [18] studied gender-related differences in cSBP and found that girls underwent a minor increase in absolute cSBP with age compared to boys. In sight of their results they suggested that these could be reflecting gender differences in growing patterns, as we discuss in the present study.

McEniery et al. [29] also reported finding higher cPP in boys than in girls with ages below 20 years, but they found a steeper increase in cPP for women. However, their results were probably influenced by including subjects with ages ranged until ~90 years (youngest ages representing a small proportion of the sample) and the existence of a marked change in VA for women with the onset of menopause is well known [36]. These results suggest that the aging rates in the youth could explain the higher cardiovascular risk for middle-aged males, whereas the process of VA during adulthood would explain an increase in elderly female risk.

On the other side, the difference in changing rate for AIx@75 seems to be beneficial for males, as we could evidence a marked decrease in this parameter. This means that at least for the first two decades of life in healthy children, adolescents, and young adults there is not a great influence of wave reflections in central BP. Our results agree with those found by Hayward and Kelly [30], Stoner et al. [37], and Ayer et al. [38], as this difference could still be found after adjusting our results by height. Two important interpretations emerge from these results: the gender difference in AIx@75 is independent of body height, and the higher levels of central BP in boys are not explained by an earlier arrival of wave reflections.

#### 4.2.2. Differences in Structural Parameters

Most of the studies investigating gender-related differences in CCA diameters and CIMT reported that boys have greater levels of CIMT, starting in postpubertal ages [20, 33]. Again, these findings together with those reporting greater CIMT levels for males in adult populations [39] determine that there must exist a gender-related difference in aging patterns to explain those differences. Engelen et al. found no gender difference in the increasing rate of CIMT, but their results could also be influenced by including individuals with a very wide range of ages. The higher increasing rate in CIMT for boys could be interpreted as an adaptative response to increasing cSBP and cPP. To test this hypothesis we calculated CCA wall stress (*σ*) for the total population and the different age groups [40]. As we could not find gender-related differences for this parameter, this result seems to back up our hypothesis.

#### 4.2.3. Differences in Arterial Stiffness

Finally, gender differences in local and regional arterial stiffness parameters have also been previously described. Ayer et al. [38] found that boys present higher local stiffness (assessed as CCA distensibility) at ~8 years of age. Several authors found that boys have higher cfPWV levels than girls. Again, these differences could be found mostly for postpubertal or transitional ages [16, 34, 41]. Elmenhorst et al. [18] also suggested the existence of different gender-related growth patterns in cfPWV, reporting a more marked increase for boys.

We found that boys have a higher increasing rate in aortic regional stiffness than girls and that this difference was BP-independent, suggesting higher levels of intrinsic wall stiffness.

### 4.3. Physiological and Clinical Implications

The marked increase in CRF exposure in pediatric ages (mostly rising prevalence of obesity and physical inactivity) in the last decades, together with accumulating evidence showing the association of early development of arterial impairment with the presence of several CRF [41–43], determines the needs for designing a new approach in order to evaluate those children at risk of developing these alterations. Moreover, evidence showing positive outcomes when carrying out early interventions in pediatric patients who already present a burden or arterial alterations [6–8] enhances the importance of early detection of vascular impairment in young individuals.

This work contributes in determining the expected growing rates for several vascular parameters in healthy individuals, free of CRF exposure. Moreover, we provide the first approach in defining the expected relative changes in arterial properties during growing and development, as normal VA may be more accurately defined by a relative amount of change in a period than by a change in absolute values.

The integral approach carried out in our study allows a more comprehensive understanding of the normal process of aging in early ages and the interaction of structural and functional parameters during this process in the same population that could be underlying the existence of a higher cardiovascular risk in adult males.

Finally, the integral description of age-related changes in arterial properties in young populations of our work could be useful for distinguishing those subjects with abnormal rates of aging in whom it would be of benefit to design proper intervention strategies.

### 4.4. Limitations

The sample size of our study was relatively small, and although several findings were statistically significant, some real differences could have been missed.

Second, for the comparison between age groups we did not determine the pubertal stage of each participant. However, the main goal of our work was to determine gender-related differences in changing rates, and the group division was made seeking to preserve at least two groups with mostly prepubertal (<8 years) and mostly postpubertal (>15 years) individuals [44].

Third, to calculate the elastic modulus, ideally the local pressure (at CCA and CFA) should be used. As the difference between aortic and CCA pressure was smaller than 2 mmHg in adults [45] central aortic pressure may be a good surrogate for CCA pressure. Local CFA pressure is in general less easy to estimate and although in this case we use brachial pressure, its values may not be the same.

Fourth, the SphygmoCor technique for the assessment of central arterial pressure uses a population-based generalized transfer function that despite being used in several studies working with children and adolescents has not yet validated for pediatric populations. Additionally, we used brachial pressure to calibrate the radial waveforms and this could lead to ignoring the potential brachial-to-radial amplification. However, we calibrate our signals utilizing brachial DBP and calculated MAP, thus minimizing possible errors. Moreover, recently Picone et al. [46] found that especially in the younger subjects (<40 years) this remains a valid way of calibrating.

Fifth, the major changes found in hemodynamic parameters could be explained on the basis of different growing-related changes in cardiac function, but parameters regarding this aspect were not assessed in the present work as this matter exceeded our main aims.

Finally, we used simple linear regression models in order to simplify our comparisons. Future works will determine the utility of using nonlinear models to describe age-related changes in arterial properties and allow determining, for example, moments of steeper changes and their differences between genders and/or with CRF exposure.

## 5. Conclusions

Gender-related differences in arterial properties found for adults can be explained on the basis of different growing-related changes between boys and girls, with no existing differences for prepubertal children. Males showed an accelerated rate of change in vascular properties (faster VA), with respect to females. Finally, we determined that the relative changes in hemodynamic parameters are higher than in structural parameters, the latter possibly being a consequence of the former.

## Figures and Tables

**Table 1:** Demographic, hemodynamic, and vascular parameters.

	Total			<8 years			8 to 14 years			≥15 years		
	Female	Male	*p*	Female	Male	*p*	Female	Male	*p*	Female	Male	*p*
	VM ± DS	VM ± DS		VM ± DS	VM ± DS		VM ± DS	VM ± DS		VM ± DS	VM ± DS	
*Anthropometric data*												
*N*	68	93		9	13		29	50		30	30	
Age (years)	16.2 ± 7.3	14.4 ± 6.2	0.08	6.8 ± 1.1	6.4 ± 0.9	0.38	11.8 ± 2.3	11.8 ± 1.9	0.93	23.4 ± 4.1	22.0 ± 3.8	0.20
Weight (kg)	46.3 ± 16.8	47.5 ± 20.8	0.69	23.2 ± 5.3	20.9 ± 2.4	0.18	40.3 ± 12.7	39.8 ± 12.1	0.85	59.4 ± 10.6	72.0 ± 9.2	**<0.001**
Height (m)	1.50 ± 0.18	1.53 ± 0.23	0.49	1.22 ± 0.08	1.17 ± 0.07	0.15	1.46 ± 0.14	1.47 ± 0.14	0.81	1.63 ± 0.07	1.77 ± 0.06	**<0.001**
Body mass index (Kg/m^2^)	19.6 ± 3.9	19.1 ± 3.7	0.44	15.5 ± 2.0	15.2 ± 1.1	0.68	18.4 ± 3.0	18.0 ± 2.6	0.51	22.2 ± 3.5	22.9 ± 2.6	0.38
*Hemodynamic parameters *												
Heart rate	79 ± 11	74 ± 12	**0.03**	87 ± 3	85 ± 11	0.65	80 ± 12	74 ± 11	0.07	75 ± 10	68 ± 12	**0.04**
Peripheral blood pressure												
Brachial SBP (mmHg)	107 ± 10	110 ± 13	0.10	98 ± 5	97 ± 7	0.77	105 ± 7	106 ± 6	0.53	114 ± 11	125 ± 11	**<0.001**
Brachial DBP (mmHg)	61 ± 7	59 ± 8	0.20	60 ± 6	57 ± 7	0.44	59 ± 6	58 ± 6	0.39	64 ± 9	64 ± 7	0.74
Brachial PP (mmHg)	46 ± 7	51 ± 10	**<0.001**	39 ± 3	42 ± 6	0.22	46 ± 6	48 ± 6	0.07	49 ± 7	60 ± 10	**<0.001**
Central blood pressure												
Aortic SBP (mmHg)	92 ± 10	95 ± 12	0.26	82 ± 6	83 ± 6	0.85	89 ± 8	90 ± 8	0.50	99 ± 10	107 ± 9	**<0.001**
Aortic DBP (mmHg)	63 ± 7	61 ± 7	0.18	61 ± 5	59 ± 7	0.43	61 ± 7	59 ± 6	0.21	68 ± 10	65 ± 8	0.19
Aortic PP (mmHg)	28 ± 6	33 ± 10	**<0.001**	21 ± 2	23 ± 5	0.34	28 ± 5	31 ± 7	**0.04**	31 ± 6	42 ± 9	**<0.001**
Aortic pulse wave analysis												
Augmented pressure (mmHg)	2 ± 3	0 ± 3	**0.01**	2 ± 2	2 ± 2	0.32	1 ± 3	0 ± 3	0.35	2 ± 3	−1 ± 4	**<0.001**
Augmentation index 75 (%)	6 ± 11	1 ± 12	**0.00**	15 ± 9	15 ± 9	0.99	6 ± 9	1 ± 10	**0.04**	6 ± 8	−8 ± 5	**<0.001**
AIx-to-height ratio	4.6 ± 7.8	1.2 ± 8.4	**0.01**	12.4 ± 7.9	12.8 ± 8.5	0.92	4.5 ± 7.4	1.0 ± 6.8	**0.04**	2.6 ± 7.1	−3.9 ± 5.2	**<0.001**
SEVR	128 ± 33	140 ± 33	**0.02**	113 ± 20	126 ± 20	0.17	119 ± 26	133 ± 32	0.05	141 ± 37	158 ± 30	0.07
*Mode B ultrasound *												
Common carotid artery												
Diastolic diameter (mm)	5.6 ± 0.5	5.9 ± 0.7	**0.02**	5.0 ± 0.4	5.2 ± 0.6	0.27	5.6 ± 0.5	5.8 ± 0.7	0.12	5.9 ± 0.4	6.3 ± 0.5	**0.01**
IMT (mm)	0.442 ± 0.046	0.459 ± 0.061	0.08	0.405 ± 0.039	0.416 ± 0.048	0.58	0.433 ± 0.036	0.449 ± 0.054	0.16	0.459 ± 0.044	0.497 ± 0.069	**0.04**
Elastic modulus (mmHg)	252 ± 100	295 ± 138	**0.05**	134 ± 28	161 ± 40	0.11	220 ± 66	267 ± 101	**0.03**	349 ± 75	424 ± 137	**0.04**
*β* Index	1.64 ± 0.22	1.75 ± 0.30	**0.02**	1.46 ± 0.15	1.50 ± 0.29	0.74	1.63 ± 0.20	1.70 ± 0.25	0.21	1.74 ± 0.21	1.98 ± 0.24	**<0.001**
Common femoral artery												
Diastolic diameter (mm)	5.6 ± 1.1	6.3 ± 1.4	**<0.001**	4.5 ± 0.8	4.6 ± 0.4	0.63	5.4 ± 0.8	6.0 ± 1.0	**<0.001**	6.8 ± 0.7	7.8 ± 0.9	**<0.001**
Elastic modulus (mmHg)	803 ± 463	877 ± 599	0.45	484 ± 198	519 ± 194	0.70	725 ± 367	723 ± 366	0.98	1146 ± 547	1386 ± 803	0.32
*β* Index	2.25 ± 0.19	2.21 ± 0.18	0.26	2.33 ± 0.17	2.37 ± 0.17	0.66	2.27 ± 0.18	2.20 ± 0.17	0.09	2.09 ± 0.16	2.11 ± 0.16	0.86
*Regional arterial stiffness *												
Carotid-femoral PWV (m/s)	4.9 ± 0.9	5.3 ± 1.1	**0.05**	4.5 ± 0.7	4.4 ± 0.6	0.88	4.7 ± 0.6	4.8 ± 0.6	0.21	5.6 ± 0.9	6.6 ± 1.0	**<0.001**
*β*PWV Index	0.62 ± 0.17	0.72 ± 0.26	**0.01**	0.56 ± 0.15	0.58 ± 0.17	0.74	0.57 ± 0.12	0.63 ± 0.15	0.10	0.75 ± 0.21	1.01 ± 0.26	**0.01**
Carotid-radial PWV (m/s)	7.5 ± 1.1	7.8 ± 1.2	0.17	7.5 ± 1.1	7.8 ± 1.5	0.65	7.4 ± 1.2	7.6 ± 1.2	0.44	7.9 ± 1.0	8.7 ± 1.2	0.07

Data expressed as mean value (M) ± standard deviation (SD). SBP, systolic blood pressure; DBP, diastolic blood pressure; PP, pulse pressure; PWV, pulse wave velocity.

**Table 2:** Age-related changes. Simple linear regression models.

	Female		*p*	Male		*p*	Slope difference
	Model	*R*		Model	*R*		*p*
*Central arterial pressure *							
Aortic SBP	0.902*x* + 77.51	0.609	<0.001	1.614*x* + 71.74	0.827	<0.001	<0.001
Aortic DBP	0.578*x* + 54.74	0.469	<0.001	0.508*x* + 53.84	0.421	<0.001	0.692
Aortic PP	0.285*x* + 23.23	0.329	0.007	1.135*x* + 17.35	0.706	<0.001	<0.001
Augmentation index 75	−0.937*x* + 18.7	0.381	0.015	−1.532*x* + 20.1	0.555	<0.001	0.202
*Mode B ultrasound*							
Carotid diastolic diameter	0.043*x* + 4.97	0.548	<0.001	0.055*x* + 5.16	0.540	<0.001	0.373
Carotid IMT	0.004*x* + 0.379	0.608	<0.001	0.006*x* + 0.378	0.575	<0.001	0.04
*Local arterial stiffness *							
Carotid elastic modulus	11.73*x* + 73.9	0.816	<0.001	16.08*x* + 75.38	0.740	<0.001	0.010
Carotid *β* Index	0.008*x* + 1.509	0.350	0.009	0.026*x* + 1.40	0.549	<0.001	0.016
Femoral elastic modulus	40.26*x* + 247.63	0.538	<0.001	58.89*x* + 70.89	0.560	<0.001	0.178
*Regional arterial stiffness *							
Carotid-femoral PWV	0.103*x* + 3.50	0.722	<0.001	0.147*x* + 3.22	0.796	<0.001	0.018
*β*PWV Index	0.016*x* + 0.39	0.585	<0.001	0.031*x* + 0.30	0.482	<0.001	0.004
Carotid-radial PWV	0.049*x* + 6.87	0.236	0.118	0.057*x* + 7.12	0.192	0.119	0.855

SBP, systolic blood pressure; DBP, diastolic blood pressure; PP, pulse pressure; IMT, intima-media thickness; PWV, pulse wave velocity.

**Table 3:** Relative change (%) predicted in arterial parameters from 4 to 18 years.

	Female				Male			
	Model	Predicted value at 4 years	Predicted value at 18 years	Relative change% (95% CI)	Model	Predicted value at 4 years	Predicted value at 18 years	Relative change% (95% CI)
*Central arterial pressure *								
Aortic SBP	0.902*x* + 77.51	81	94	15.6 (10.7–20.35)	1.614*x* + 71.74	78	101	28.9 (25.0–32.7)
Aortic DBP	0.578*x* + 54.74	57	65	14.2 (7.7–20.5)	0.508*x* + 53.84	56	63	12.7 (7.1–18.2)
Aortic PP	0.285*x* + 23.23	24	28	16.4 (4.8–27.2)	1.135*x* + 17.35	22	38	72.6 (59.9–84.2)
Augmentation index 75	−0.937*x* + 18.7	15	2	88 (15–197)	−1.532*x* + 20.1	14	−7	153 (87–244)
*Mode B ultrasound*								
Carotid diastolic diameter	0.043*x* + 4.97	5.14	5.74	11.7 (7.2–16.1)	0.055*x* + 5.16	5.38	6.15	14.3 (9.5–18.7)
Carotid IMT	0.004*x* + 0.379	0.395	0.451	14.2 (10.7–20.8)	0.006*x* + 0.378	0.402	0.486	20.9 (14.2–27.3)
*Local arterial stiffness *								
Carotid *β* Index	0.008*x* + 1.509	1.54	1.65	7.3 (0.9–15.1)	0.026*x* + 1.40	1.50	1.87	24.2 (16.2–31.8)
Carotid elastic modulus	11.73*x* + 73.9	121	285	135.9 (116.3–152.5)	16.08*x* + 75.38	140	365	161.1 (139.8–178.5)
Femoral elastic modulus	40.26*x* + 247.63	409	972	137.9 (93.3–169.3)	58.89*x* + 70.89	306	1131	269 (241–286)
*Regional arterial stiffness *								
Carotid-femoral PWV	0.103*x* + 3.50	3.91	5.35	36.9 (27.9–45.6)	0.147*x* + 3.22	3.81	5.87	54.0 (46.4–61.6)
*β*PWV Index	0.016*x* + 0.39	0.45	0.68	49.3 (32.6–66.8)	0.031*x* + 0.30	0.45	0.98	102.4 (84.8–117.7)

SBP, systolic blood pressure; DBP, diastolic blood pressure; PP, pulse pressure; IMT, intima-media thickness; PWV, pulse wave velocity.

**Table 4:** Regression models for cfPWV, CIMT, and cSBP (as a function of age) from other authors.

	Male	Female
cfPWV		
This paper	0.147*x* + 3.22	0.103*x* + 3.50
Reusz et al. [16]	0.121*x* + 3.356	0.089*x* + 3.749
Fischer et al. [17]	0.133*x* + 3.108	0.080*x* + 3.468
Elmenhorst et al. [18]	0.088*x* + 3.588	0.051*x* + 3.936
CIMT		
This paper	0.006*x* + 0.378	0.004*x* + 0.379
de Arriba Muñoz et al. [19]	0.009*x* + 0.276	0.010*x* + 0.261
Doyon et al. [20]	0.003*x* + 0.347	0.001*x* + 0.357
cSBP		
This paper	1.614*x* + 71.74	0.902*x* + 77.51
Elmenhorst et al. [18]	2.206*x* + 72	1.456*x* + 80.1

cfPWV, carotid-femoral pulse wave velocity; CIMT, carotid intima-media thickness; cSBP, central systolic blood pressure.
